# Decreased risk of non-influenza respiratory infection after influenza B virus infection in children

**DOI:** 10.1017/S0950268824000542

**Published:** 2024-04-08

**Authors:** Tim K. Tsang, Richael Q. R. Du, Vicky J. Fang, Eric H. Y. Lau, Kwok Hung Chan, Daniel K. W. Chu, Dennis K. M. Ip, J. S. Malik Peiris, Gabriel M. Leung, Simon Cauchemez, Benjamin J. Cowling

**Affiliations:** 1 WHO Collaborating Centre for Infectious Disease Epidemiology and Control, School of Public Health, Li Ka Shing Faculty of Medicine, The University of Hong Kong, Hong Kong; 2 Laboratory of Data Discovery for Health Limited, Hong Kong Science and Technology Park, Hong Kong; 3 Department of Microbiology, Li Ka Shing Faculty of Medicine, The University of Hong Kong, Hong Kong; 4 HKU-Pasteur Research Pole, The University of Hong Kong, Hong Kong; 5 Centre for Immunology and Infection, Hong Kong Science and Technology Park, Hong Kong; 6 Mathematical Modelling of Infectious Diseases Unit, Institut Pasteur, UMR2000, CNRS, Paris, France

**Keywords:** acute respiratory illness, children, influenza, non-influenza respiratory virus, non-influenza respiratory virus infection, temporary protection, virus interference

## Abstract

Previous studies suggest that influenza virus infection may provide temporary non-specific immunity and hence lower the risk of non-influenza respiratory virus infection. In a randomized controlled trial of influenza vaccination, 1 330 children were followed-up in 2009–2011. Respiratory swabs were collected when they reported acute respiratory illness and tested against influenza and other respiratory viruses. We used Poisson regression to compare the incidence of non-influenza respiratory virus infection before and after influenza virus infection. Based on 52 children with influenza B virus infection, the incidence rate ratio (IRR) of non-influenza respiratory virus infection after influenza virus infection was 0.47 (95% confidence interval: 0.27–0.82) compared with before infection. Simulation suggested that this IRR was 0.87 if the temporary protection did not exist. We identified a decreased risk of non-influenza respiratory virus infection after influenza B virus infection in children. Further investigation is needed to determine if this decreased risk could be attributed to temporary non-specific immunity acquired from influenza virus infection.

## Introduction

Virus interference describes the phenomenon that an infection for a pathogen may have impact on subsequent infection of other pathogens. It has been observed in many diseases for decades and first identified in virologic studies [1–4]. Ecological studies usually suggested virus interference by negative association between incidences of diseases, including measles and whooping cough [5]. This is particularly studied for respiratory virus, with a focus on interference between influenza and other respiratory virus, including respiratory syncytial virus (RSV) [6–8], parainfluenza virus [9], adenovirus [9], and rhinoviruses [10, 11]. This association has also been observed among other non-influenza respiratory viruses, such as between rhinoviruses and adenoviruses [12] and between RSV and rhinoviruses [13]. However, positive association between incidences of disease is also possible, such as the change of testing capacity [14], or population-level interventions such as social distancing measure that prevented spread for respiratory viruses [15], so that the observed number of respiratory virus infections increased or decreased simultaneously.

One potential mechanism for the negative association between incidence of disease was that a viral infection may provide a temporary non-specific immunity against infection of another virus [16, 17]. If this is true, then vaccination against a virus may decrease the risk of natural infection of that virus, which would otherwise provide temporary non-specific immunity against other viruses, and hence increase the risk of infection of other viruses. One example is influenza and other respiratory viruses, which was reported in a vaccine trial in 2008/09 [16]. Live attenuated influenza vaccines [18] and live attenuated polio vaccines [19, 20] have been reported to provide temporary non-specific protection against other infections, presumably through the same mechanism [21].

Here, we analysed data from a randomized controlled trial of influenza virus vaccination in 2009/10, with a follow-up to identify virologically confirmed influenza and other respiratory virus infections [22]. We compared the incidence of non-influenza respiratory virus infections before and after influenza B virus infection.

## Methods

### Study design

In 2009/10, we conducted a community-based randomized controlled trial to evaluate the direct and indirect benefits of influenza vaccination (ClinicalTrials.gov NCT00792051). We enrolled households that each included at least one child aged 6–17 years. In each household, one child was randomized to receive either a single dose of trivalent inactivated influenza vaccination (0.5 mL of VAXIGRIP; Sanofi Pasteur) or saline placebo [22]. Telephone calls were made every 2 weeks to monitor for any acute upper respiratory tract infections. Home visits were triggered when any household member reported the presence of any of the following two symptoms: fever ≥37.8°C, chills, headache, sore throat, cough, presence of phlegm, coryza, or myalgia. Additional visits were conducted at 3-day intervals until acute illnesses resolved. In each home visit, nasal and throat swab specimens were collected from all household members, regardless of presence of illness, for laboratory testing. Participants were followed up with the same design for 2010/11.

All participants aged 18 years and older gave written informed consent. Proxy written consent from parents or legal guardians was obtained for participants, with additional written assent from those aged 8–17 years. The study protocol was approved by the Institutional Review Board of the University of Hong Kong.

Study participants who met the eligibility criteria were randomly assigned to either the trivalent inactivated vaccine (TIV) or placebo group, following a 3:2 allocation ratio. To maintain blinding for both households and study nurses, a trained nurse who was not involved in administering the vaccines repackaged TIV and placebo into identical, numbered syringes. A research assistant, without access to the randomization list, assigned unique identification numbers to the participating households based on the order in which they attended. These identification numbers were then matched to the vaccine packages. The allocation of TIV or placebo remained concealed to the households, study nurses, and laboratory staff, and was disclosed to the investigators only upon the completion of the follow-up period.

### Laboratory methods

Pooled nose and throat swab samples were stored at −80˚C and tested for influenza A and B by reverse-transcription polymerase chain reaction (RT-PCR), using standard methods as described elsewhere [16]. The swab samples collected before February 2011 were also tested for 19 non-influenza respiratory viruses by the ResPlex II Plus multiplex array as described elsewhere [16].

### Statistical methods

Since almost all (>98%) non-influenza respiratory virus infections in our study occurred in children (aged 0–17), adult household members (aged ≥18) were excluded in our analysis. Influenza virus infections were defined as a positive PCR result on testing of one or more pooled nasal and throat specimen collected from that individual. We focused on children with influenza B virus infections since this type was prevalent during the study period and the trivalent inactivated influenza vaccine was estimated to have 66% efficacy against influenza virus infections in our trial [22]. We considered that the follow-up ended at 28 February 2011 or earlier if loss of follow-up, since the swab samples collected after that day were not tested against non-influenza respiratory viruses.

Among those individuals with influenza virus infection, we used a self-controlled approach to estimate the incidence rate ratio (IRR) of non-influenza respiratory infections after and before influenza virus infection. Given that there were seasonal patterns of influenza and other non-influenza respiratory viruses, we used a piecewise Poisson regression model that assumed the risk was constant in each week but may differ between weeks, with an offset term for the person-time before and after infections. This approach was used to address the potential individual differences such as differences in reporting after presence of symptoms, health status, and heterogeneity in exposure [23].

To validate that this approach was robust to the reporting difference between individuals with or without influenza virus infections, we conducted the same analysis on the individuals without influenza virus infection, by randomly imputing a ‘virtual’ infection time from the observed time of influenza virus infection in the data, to estimate the ‘null’ IRR among individuals without influenza virus infection and hence without temporal protection. As sensitivity analyses, we conducted the same simulations, but randomly imputing a ‘virtual’ infection time from the observed time of influenza virus infection in the data, but added 3 and 6 months (so that there should be no temporal protection) to determine if the impact of different timing of influenza seasons when estimating the ‘null’ IRR. Statistical analyses were conducted using R version 3.5.2 (R Foundation for Statistical Computing, Vienna, Austria).

## Results

From August 2009 to February 2010, we enrolled 796 households, each of which included one child that was randomly allocated to receive influenza vaccination or placebo, and an additional 534 household contacts aged between 1 and 17 years. One child withdrew from the study after randomization but before the intervention was administered, and 13 of the 795 children who received the intervention did not complete the study. Other participants were followed up through October 2010, but 193 households did not join the second year of the study. To account for this, Poisson regression with offset of the duration of follow-up was used.

We detected 13/479 [3%], 23/317 [7%], and 16/534 [3%] PCR-confirmed influenza B virus infections in children randomized to vaccination, placebo, and child household contacts, respectively. During the study period, we observed the following non-influenza respiratory virus infections: rhinovirus, metapneumovirus, coronavirus, parainfluenza virus, RSV, and adenovirus (Table 1). Rhinovirus was responsible for over half of the non-influenza respiratory virus infections, accounting for 70% (310/448) of cases. Among children who were randomized to receive either the vaccine or placebo, as per the study design (Table A1), the incidence of influenza virus infection in the placebo group (0.06 per person-year; 95% confidence interval (CI): 0.04–0.10) was greater than that in the TIV group (0.02 per person-year; 95% CI: 0.01–0.04; *p* = 0.005). This result indicated a vaccine effectiveness against PCR-confirmed influenza of 63% (95% CI: 26%–81%).

**Table 1.** Incidence rates of respiratory virus detection by reverse-transcription polymerase chain reaction and X-Tag multiplex assay by infection status of influenza B virus infection

	Before influenza B virus infection			After influenza B virus infection			Without influenza B virus infection		
	Incidence rate			Incidence rate			Incidence rate		
	No.	Rate	(95% CI)	No.	Rate	(95% CI)	No.	Rate	(95% CI)
Person–years	20.84			40.98			1 423.85		
Any non–influenza virus	**25**	**1.20**	**(0.81–1.78)**	**23**	**0.56**	**(0.37–0.84)**	**400**	**0.28**	**(0.25–0.31)**
Rhinovirus	14	0.67	(0.4–1.13)	17	0.41	(0.26–0.67)	279	0.2	(0.17–0.22)
Metapneumovirus	5	0.24	(0.1–0.58)	1	0.02	(0–0.17)	33	0.02	(0.02–0.03)
Coronavirus	0	0	NA	2	0.05	(0.01–0.2)	33	0.02	(0.02–0.03)
Parainfluenza	1	0.05	(0.01–0.34)	2	0.05	(0.01–0.2)	25	0.02	(0.01–0.03)
Respiratory syncytial virus	4	0.19	(0.07–0.51)	1	0.02	(0–0.17)	20	0.01	(0.01–0.02)
Adenovirus	1	0.05	(0.01–0.34)	0	0	(0–Inf)	10	0.01	(0–0.01)

Significance level is 5%.

**Figure 1.**
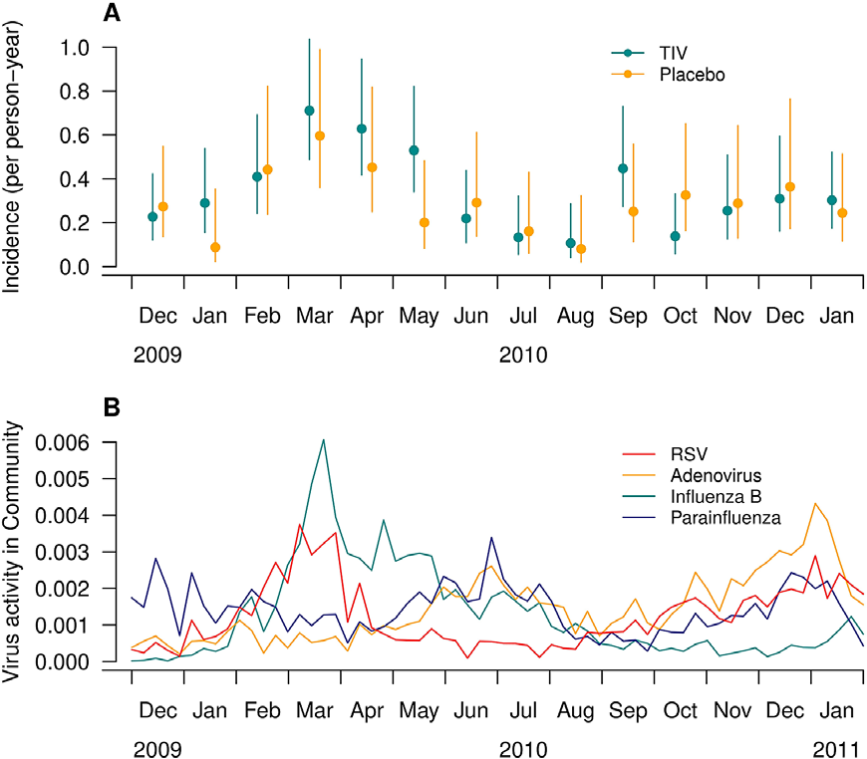
Monthly incidence of non-influenza respiratory virus infections for the study period. (A) The red and orange points and lines indicated the incidence and the corresponding 95% confidence interval of non-influenza respiratory virus infection for children in vaccine (TIV) and placebo groups, respectively. (B) The red, orange, deep green, and purple lines showed the RSV, adenovirus, influenza B, and parainfluenza virus activities based on surveillance data.

Monthly incidence of non-influenza respiratory virus infections for the children who received influenza vaccination or placebo are shown in Figure 1. While non-influenza virus activity (blue line) was stable over the year, we noted that the largest difference in the incidence of non-influenza respiratory virus infections between vaccine recipients and placebo recipients was observed in May 2010, that is, 1–2 months after the peak of influenza B virus activity (black line) in the community (March to April 2010). Overall, the incidence of non-influenza virus infection for children who received influenza vaccination was slightly higher than for those in the placebo group, but the difference was not statistically significant (IRR: 1.18; 95% CI: 0.92–1.51; *p*-value: 0.20).

Incidence of non-influenza respiratory virus infections before and after influenza virus infection were 1.20 (95% CI: 0.81–1.78) and 0.56 (95% CI: 0.37–0.84) per person-year, respectively. The incidence of non-influenza respiratory virus infections in these two groups were much higher than in those without influenza virus infection (incidence rate: 0.28; 95% CI: 0.25–0.31). As described earlier, we hypothesized that the lower incidence of non-influenza respiratory virus infection in individuals without influenza virus infection was due to difference in reporting behaviour (mean episode of ILI reported: 2.63 and 0.86 for individuals with and without influenza virus infection, respectively). Therefore, we used a self-controlled approach to test if this association was robust to such differences.

Based on those individuals with influenza virus infection, the IRR of non-influenza respiratory virus infection after and before influenza virus infection was 0.54 (95% CI: 0.30–0.963, *p*-value: 0.037), suggesting a temporal protection against non-influenza respiratory virus infection after influenza virus infection. In the validation analysis with 10 000 replications by randomly selecting 200 000 individuals (with replacement) without influenza virus infection and hence no temporal protection, and randomly assigning the ‘infection time’ based on the observed infection time, this ‘null’ IRR was 0.976. We repeated the same analysis, by randomly selecting 200 000 individuals (with replacement) without influenza virus infection, and randomly assigning the ‘infection time’ based on the observed infection time, but added 3 and 6 months, and these ‘null’ IRR were 1.035 and 1.024. Both ‘null’ IRRs were higher than the observed one, indicating that the decreased risk could not be explained by reporting differences alone.

## Discussion

In this study, we used a self-controlled analysis to identify a reduction in the incidence rate of non-influenza respiratory virus infections (IRR: 0.54; 95% CI: 0.30–0.963) after PCR-confirmed influenza B virus infections. In a validation analysis, we estimated that among those individuals without influenza virus infection and hence without temporal protection, the ‘null’ IRR was 0.976. We conducted different sensitivity analyses to estimate this ‘null’ IRR, and the estimates ranged from 1.024 to 1.035. Hence, these results suggested that this difference cannot be explained by reporting differences among individuals with or without influenza virus infections.

This decreased risk of non-influenza respiratory virus infection after influenza virus infection is consistent with the potential for temporary non-specific immunity, due to the innate immune response that is triggered by one viral infection and protects against all viral infections for a short time [24]. We postulate that the risk of non-influenza respiratory virus infections after influenza virus infections was decreased due to temporary non-specific immunity after the influenza virus infection.

The reduced risk of non-influenza respiratory virus infection following influenza virus infection observed in our study at the individual level aligns with findings in ecological studies conducted at the population level. For instance, some studies have noted that influenza outbreaks may postpone the RSV season [6] and that there is asynchronous circulation between influenza and rhinovirus [10, 11]. Our results support the inverse relationship between the incidence of influenza virus and non-influenza respiratory virus infections observed in ecological studies, suggesting that this relationship is not solely attributable to surveillance bias [11]. Gaining a deeper understanding of this phenomenon is crucial for accurately characterizing the epidemiological dynamics of influenza and other respiratory viruses. Such knowledge may also contribute to enhancing disease forecasting models [11]. During the COVID-19 pandemic, there has been a significant decrease in the activity of certain respiratory viruses, including the influenza virus. However, this reduction is likely due to the implementation of stringent public health measures, such as lockdowns, which have decreased the transmission of various pathogens, rather than being a result of virus interference.

In our study, we observed a higher, albeit not statistically significant, risk of non-influenza respiratory virus infections among vaccinated participants compared to non-vaccinated participants throughout the study period. This risk increased more than fourfold in a smaller influenza vaccination trial conducted in Hong Kong during 2008/09 [16], but to a lesser degree in a test-negative design study among army personnel in the United States [25]. Our analysis indicates that this phenomenon likely resulted from the absence of temporary non-specific immunity following influenza virus infection, such as the innate immune response to infection [24]. This hypothesis aligns with the observation that the largest difference in the incidence of non-influenza respiratory virus between participants in the vaccine group and the placebo group occurred in May 2010, approximately 1 month after the peak of influenza B (Figure 1). It is plausible that receiving the influenza vaccine may directly increase the risk of non-influenza respiratory virus infection due to an unknown biological mechanism, such as enhancing immunity against influenza while concurrently reducing immunity against other respiratory viruses.

Our study has some limitations. First, we combined several respiratory viruses together, to form a group of ‘non-influenza respiratory viruses’ infections, due to a lack of sample size. However, most literature suggested that virus interference between influenza virus and various non-influenza respiratory virus infection were similar [6]. Second, we only observed one PCR-confirmed non-influenza respiratory virus infection in adults. Therefore, we were unable to examine whether virus interference might also occur in adults, although it may be expected that the effect is smaller because the risk of influenza virus infection and non-influenza respiratory virus infections are also lower than children. Third, we cannot rule out the potential of other unidentified confounders in association between the incidence of influenza virus and non-influenza respiratory virus infection. For example, there could be measurement bias that participants were more likely to report their first illness episode, but not the subsequent episodes. Because the reporting frequency among individuals differed by threefold, we adopted a self-controlled approach that compared the incidence of non-influenza respiratory virus infection before and after an influenza virus infection [23, 26]. Fourth, we used piecewise Poisson regression that assumed the risk within a week was the same, but it could differ between weeks, to account for the seasonality of influenza and non-influenza respiratory viruses. While we could divide the period to a smaller scale like day, the number of events in our study did not allow for smaller scale. Finally, the self-controlled approach we use could not determine the duration of temporary non-specific immunity, we noted that the hypothesized potential temporary immunity induced by influenza infection is short-term, and a relatively short post-influenza infection window would be most relevant.

In conclusion, we found a reduction in risk of infection with non-influenza respiratory viruses after influenza B virus infection. This is consistent with temporary non-specific immunity which is one of the biological mechanisms proposed to explain virus interference at the ecological level.

## Data Availability

The data that support the findings of this study are openly available at https://github.com/bcowling/pediatric-vaccine-trial/tree/master/data.

## Appendix

**Table A1.** Incidence rates of respiratory virus detection by reverse-transcription polymerase chain reaction and X-Tag multiplex assay

	TIV			Placebo			Household contacts		
	Incidence			Incidence			Incidence		
	No.	Rate	(95% CI)	No.	Rate	(95% CI)	No.	Rate	(95% CI)
*N*	479			317			534		
Influenza virus	**13**	**0.02**	**(0.01–0.04)**	**23**	**0.06**	**(0.04–0.10)**	**16**	**0.03**	**(0.02–0.04)**
Any non–influenza virus	**174**	**0.33**	**(0.28–0.38)**	**98**	**0.28**	**(0.23–0.34)**	**176**	**0.29**	**(0.25–0.34)**
Rhinovirus	122	0.23	(0.19–0.27)	66	0.19	(0.15–0.24)	122	0.2	(0.17–0.24)
Metapneumovirus	12	0.02	(0.01–0.04)	7	0.02	(0.01–0.04)	20	0.03	(0.02–0.05)
Coronavirus	15	0.03	(0.02–0.05)	10	0.03	(0.02–0.05)	10	0.02	(0.01–0.03)
Parainfluenza	12	0.02	(0.01–0.04)	6	0.02	(0.01–0.04)	10	0.02	(0.01–0.03)
Respiratory syncytial virus	6	0.01	(0.01–0.03)	7	0.02	(0.01–0.04)	12	0.02	(0.01–0.04)
Adenovirus	7	0.01	(0.01–0.03)	2	0.01	(0–0.02)	2	0	(0–0.01)

Significance level is 5%.
